# Psychometric properties of the Indonesian Ten-item Internet Gaming Disorder Test and a latent class analysis of gamer population among youths

**DOI:** 10.1371/journal.pone.0269528

**Published:** 2022-06-14

**Authors:** Kristiana Siste, Enjeline Hanafi, Lee Thung Sen, Reza Damayanti, Evania Beatrice, Raden Irawati Ismail

**Affiliations:** Department of Psychiatry, Faculty of Medicine, Universitas Indonesia–dr, Cipto Mangunkusumo General Hospital, Jakarta, Indonesia; Sapienza, University of Rome, ITALY

## Abstract

Internet gaming disorder (IGD) is a rising health concern. Indonesia has yet to have any validated instrument specifically designed to screen for this disorder. This study aims to validate the Indonesian version of the Ten-item Internet Gaming Disorder Test (IGDT-10) and conduct a latent class analysis of gamers among the youth. An online survey was conducted between October and December 2020 at two universities in Depok and Jakarta, Indonesia. In total, 1233 respondents (62.6% female and 20.3±1.90 years old) gave valid responses and played video games. Confirmatory factor analysis (CFA) confirmed the unidimensional structure of the scale. Cronbach’s alpha was 0.72 and composite reliability was 0.92. The latent class analysis yielded three distinct classes of gamers. The continuation and negative consequences were highly distinctive for the group at high risk of IGD (class 3). Deception had the lowest endorsement rate (41.7%); while, the continuation domain had the highest endorsement, 91.2%. The IGD prevalence estimate was 1.90% among the respondents. Approximately 70.2% of the gamers did not show IGD symptoms. The adapted Indonesian IGDT-10 was demonstrated as valid and reliable among Indonesian youths. Consistent with previous studies, the deception domain had a low endorsement rate. The detected IGD rates were comparable to the global range. The majority of the current sample disclosed no symptoms; however, a considerable proportion would benefit from early preventive measures.

## Introduction

Internet gaming disorder (IGD) is defined as continuous and repetitive immersion in gaming, either alone or with others, which causes clinically significant impairment or distress [1]. Further, pathological use can develop from both offline and online games [2]. The inclusion of IGD in Section III of the fifth edition of the Diagnostic and Statistical Manual of Mental Disorders (DSM-5) has significantly increased its awareness and research. A previous related study reported that the prevalence of IGD ranged from 0.7–15.6% in the general population [3]. Another meta-analysis noted a pooled prevalence of IGD of 10.1% among adolescents in Southeast Asia [4]. The increase in prevalence in recent years could be attributed to the rising popularity of online games, with an estimated 1 billion gamers worldwide in 2012 and reaching 2.7 billion in 2020 [5]. Multiple related studies have indicated the negative consequences of IGD, such as physical complaints (sleep deprivation, reduced nutritional status, and muscle soreness) and psychosocial morbidities, such as diminished real-life relationship quality, lost opportunities, depressive symptoms, and aggression [6, 7]. Therefore, it is paramount for physicians to recognize and screen for IGD in the early phase of the disease. To that end, a formal diagnosis similar to IGD was recently formalized by the World Health Organization (WHO) [8].

According to the DSM-5, IGD encompasses nine diagnostic criteria: (i) developing preoccupation with games, (ii) experiencing withdrawal symptoms, (iii) developing tolerance of gaming time, (iv) lack of control while gaming, (v) losing interest in other hobbies or activities, (vi) continuous involvement in gaming despite adverse effects, (vii) lying about gaming time, (viii) using Internet gaming to avoid negative feelings or improve mood, and (ix) losing relationships or opportunities in education or career [1]. To help diagnose IGD, numerous screening instruments have been developed [9], among which is the ten-item Internet gaming disorder test (IGDT-10) developed by Király et al. [10] according to the nine diagnostic criteria in DSM-5. This questionnaire has been validated across multiple languages [10–14]. This instrument comprises 10 items; in which item 1 to 8 each represents a single IGD diagnostic criterion, while item 9 and 10 jointly identify the negative consequences domain [10]. Some of the superior qualities of IGDT-10 are its conceptual specificity, brevity, and practicality to be conducted in a large-scale survey or clinical practice. Despite the term IGD, the DSM-5 outlines that the disorder also comprises non-Internet computerized games (e.g., video games) [1], which is also operationalized in IGDT-10 [10]. A recent review demonstrated that the IGDT-10 also fulfilled the criteria set out by WHO for gaming disorder in its International Classification of Diseases, 11^th^ Revision [8]. The WHO gaming disorder criteria encompasses: (i) impaired control, (ii) increasing priority, (iii) continuation of gaming, and (iv) significant impairment [15].

While it is crucial to identify IGD, there is no validated instrument in Indonesia to assess and screen the risk of the disorder. ICD-11 sets out three main criteria to diagnose gaming disorder, all of which are contained in the DSM-5 and employed by IGDT-10 thus allowing for a more detailed scrutiny on a novel population. Currently, ICD-11 is awaiting field testing finalization for its applicability in Indonesia. Notably, IGDT-10 had been previously validated against clinical diagnosis in China [12]. Taking these points into account, the current study aims to translate and adapt the English version of the IGDT-10 into the Indonesian language and validate its psychometric properties among the youth. This study also scrutinizes the latent classes of the gamer population, compares the IGD prevalence estimates with those indicated in other studies, and analyzes its associated factors among the sample. The adapted IGDT-10 could be used for screening in broader public or healthcare settings in Indonesia.

## Materials and methods

### Participants and procedure

The respondents were recruited from University X (public) in Depok and University Y (private) in Jakarta, Indonesia. Faculties were randomly selected from clusters of health sciences, social sciences, and natural sciences at each university. All the faculties accepted the invitation to participate in this study. Letters were sent to each participating faculty. The hyperlink was shared through all the faculties’ online boards, faculty administrators, and student bodies from October to December 2020. Students who had gaming experience within the past year were invited.

This study used Google form as a third-party online tool. The respondents were presented with a description of the research and measures to secure private data upon clicking the link. They were further reminded that participation was voluntary, provided with an e-mail address for further inquiry, and asked to provide electronic written informed consent before participating. The survey encompassed sociodemographic factors, gaming-related variables, and the translated Ten-Item Internet Gaming Disorder Test (IGDT-10). All the items were marked as mandatory to avoid missing data. The survey was estimated to take 15 minutes. Overall, 1265 respondents gave valid responses; however, 32 reported to not playing any video games in the past year and were omitted from detailed analyses. Specifically, 1080 came from the health sciences, 149 from natural sciences, and 36 from social sciences.

### Measures

#### Sociodemographic characteristics

The same online questionnaire was administered to all the IGDT-10 samples. Data on age, sex, and residence were collected.

#### Gaming-related variables

The respondents were asked to fill in data related to their habit of playing games, such as the age of first gaming, weekday gaming duration, weekend day gaming duration, main gaming platform, gaming community, the purpose of playing games, game genres, and negative consequences. The age of first gaming, weekday gaming duration, and weekend day gaming duration were presented in the numerical data. The gaming community was evaluated as a nominal variable with only two response options: “yes” and “no.” The response options for main gaming platform were as follows: (1) mobile phones, (2) PC/desktop, (3) laptop, and (4) tablet. The purpose of playing games was categorized as (1) entertainment, (2) pastime, (3) relieve stress, (4) achievement, and (5) socialize. The game genres options were comprised of first-person shooting (FPS), battle royale, simulation games, sports, multiplayer online battle arena (MOBA), real-time strategies, massively multiplayer online role-playing games (MMORPG), puzzle, and fighting. The negative consequences included disrupted sleep patterns, work/academic related issues (whether participants perceived experiencing problems in their work or school due to gaming), physical symptoms, poor eating patterns, mood problems, relationship related problems, weight gain, and hostility. These variables were applicable for all respondents, either playing online or offline games more; although, certain sub-options might be relevant only for online gaming (e.g., game genres of MOBA and MMORPG).

#### The Ten-Item Internet Gaming Disorder Test (IGDT-10)

IGDT-10 was developed by representing each of the nine criteria of IGD within DSM-5 through an item, except for the impairment domain that is represented by two items (item 9 and 10) [10]. The tool adopts a 3-point Likert scale (0 = never, 1 = sometimes, 2 = often). To mimic DSM-5’s dichotomous nature, scores of zero and one are coded as 0 (= no, suggesting that the behavior or problems are not frequent), and scores of two are coded as 1 (= yes, suggesting the behavior or problems are frequent or continuously present). A response of "Often" on either item nine or ten (or both) is considered one point as they represent a single construct (negative consequences). The summed score ranges from zero to nine, with a cutoff score of five.

### Translation procedure

First, the research team e-mailed the original author to procure the English version of IGDT-10, which was forward translated by two separate independent certified translators who had not seen the instrument and were bilingual. One of the translators understood medical lexicons, while the other did not. The two resulting versions were then discussed among a panel of experts comprising an addiction psychiatrist, a neuropsychiatrist, and a child and adolescent psychiatrist. The translated and merged IGDT-10 was reverse translated by a separate certified independent translator, who was also bilingual and had not seen the instrument. The back-translation was e-mailed to the original author for comparison with the original in terms of accuracy and consistency in meaning.

### Statistical analyses

The following statistical analyses were performed: (1) descriptive characteristics among the entire sample, the sample with those that play video games (video game players), and those that do not play video games (non-video game players); (2) confirmatory factor analysis (CFA) of the construct validity of the Indonesian version of IGDT-10; (3) evaluation of the internal reliability of the Indonesian IGDT-10 scale using Cronbach’s alpha and composite reliability; (4) an estimation of the proportion of IGD in the studied population; (5) a latent class analysis (LCA) of gamers within the youth sample; (6) endorsement rate for the IGD-risk and total sample group; (7) statistical differences (chi-square or Fisher’s exact test and independent T-test) between IGD-risk and healthy respondents; and (8) correlation analysis (Pearson or Spearman) between total raw scores of IGDT-10 with age and gaming-related variables. For analysis (7), 100 samples, each comprising 24 participants were randomized from the healthy respondent pool (N = 1209), averaged, and then compared to the IGD-risk group of a similar size (N = 24). All analyses were conducted using the IBM SPSS Statistics version 22, R version 4.1.2, and Mplus version 8.3 statistical packages.

The confirmatory factor analysis (CFA) was used to test the dimensionality and construct validity of the IGDT-10. The model estimation utilized weighted least square mean and variance (WLSMV) in *lavaan* package version 0.6–9. The nine IGDT domains for WLSMV were treated as ordinal and all variables and each possible option (never/sometimes/often) were reported by at least a single respondent. The composite reliability was calculated using the formula by Raykov [16]. The model fit followed the criteria set out by Hu and Bentler [17], root mean square error of approximation (RMSEA) < 0.06, comparative fit index (CFI) ≥ 0.90, standardized root mean square residual (SRMR) <0.08, Tucker-Lewis index (TLI)/non-normed fit index (NNFI) > 0.95, and adjusted goodness-of-fit index (AGFI) > 0.95. Statistical significance was set at *P* < 0.05. The resulting factors were then analyzed for internal reliability (Cronbach’s alpha and composite reliability) and items convergence (item total correlations).

Latent class analysis (LCA) was performed using the Mplus version 8.3 imposing the default restrictions (e.g., local independence). The nine dichotomized variables of the IGDT-10 corresponding to the nine IGD diagnostic criteria proposed by the DSM-5 were used as indicators for class clustering. The optimal number of latent classes was determined by scrutinizing lower values of the Bayesian Information Criterion (BIC), sample-size adjusted BIC (ABIC), Akaike Information Criterion (AIC), and *p*< 0.05 for the Lo-Mendell-Rubin Adjusted Likelihood Ratio Test (LMR) and the Parametric Bootstrapped Likelihood Ratio Test [18]. The endorsement rate was evaluated for the IGD-risk and total sample group and computed as the number of participants who answered yes for each diagnostic criterion divided by the total number of subjects in each group. IGD-risk group was comprised of participants who had IGDT-10 total score ≥ 5.

### Ethics

The study was approved by the Institutional Review Board of the Faculty of Medicine, Universitas Indonesia (No: KET-885/UN2.F1/ETIK/PPM.00.02/2020). Electronic written informed consent was obtained from all respondents.

## Results

### Participants descriptive

A total of 1265 respondents volunteered to join this study. Table 1 describes the characteristics of the entire sample, the sample with those that play video games (video game players), and those that do not play video games (non-video game players, N = 32). In this study, the proportion of respondents who played video games was 97.5% (N = 1233). Only those who played video games were used for calculating the statistical analyses.

**Table 1:** Respondents’ descriptive between video game players and non-video game players.

Variable		Video Game Player (N = 1233)	Non-Video Game Player (N = 32)	Total (N = 1265)
		M±SD/N(%)	M±SD/N(%)	M±SD/N(%)
**Age**		20.3±1.9	19.3±1.8	20.3±1.9
**Sex**				
	**Male**	470 (38.1)	4 (12.5)	474 (37.4)
	**Female**	763 (61.9)	28 (87.5)	791 (62.6)
**Residence**				
	**Without family**	555 (45.0)	13 (45.0)	568 (44.9)
	**With family**	678 (55.0)	19 (55.0)	697 (55.1)

### Factor structure, reliability, and validity

**Fig 1:**
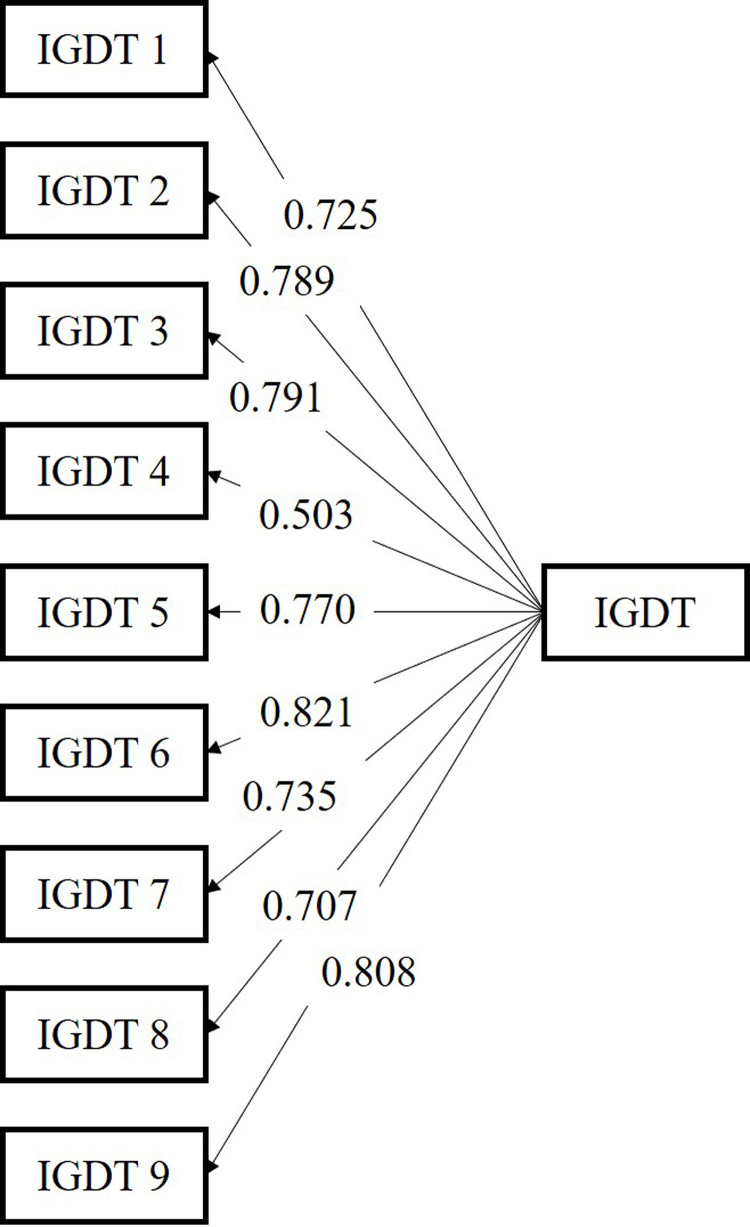
CFA results of the one-factor structure of the Indonesian IGDT-10. Values represent standardized factor loadings. IGDTs 1 to 9 represent each domain of the IGD. IGDT-10, Ten-item Internet Gaming Disorder Test.

The constructs within the IGD were proposed as a one-factor solution based on previous empirical and theoretical evidence [10]. The uni-dimensionality of the Indonesian IGDT-10 was analyzed using CFA, and all items loaded significantly on a single latent factor according to the standardized factor loadings (see Fig 1). The model also demonstrated satisfactory fit indices, **χ**2 (df = 27, *P* = 0.034) = 41.92 and **χ**2 /df = 1.55, RMSEA = 0.067, CFI = 0.989, SRMR = 0.021, TLI = 0.985, and AGFI = 0.994.

The overall Cronbach’s alpha value was 0.72. The corrected item-total correlation was used to identify non-convergent items. Item 4 had a total item correlation value less than 0.30. Upon further scrutiny, its deletion reduced the internal reliability; as such, it was retained (Table 2). The value of composite reliability for the IGDT-10 in this study was 0.92.

**Table 2:** Standardized factor loading and reliability indicators.

Item	Standardized Factor Loading	Corrected Item-Total Correlation	Cronbach’s Alpha if Item Deleted
**1. Preoccupation**	0.725	0.398	0.661
**2. Withdrawal**	0.789	0.399	0.667
**3. Tolerance**	0.791	0.450	0.649
**4. Loss of control**	0.503	0.250	0.694
**5. Loss of interest**	0.770	0.398	0.664
**6. Continuation**	0.821	0.489	0.641
**7. Deception**	0.735	0.330	0.678
**8. Escape**	0.707	0.399	0.676
**9. Negative consequences**	0.808	0.379	0.673

### Latent class analysis, criteria endorsement, and prevalence estimate

**Fig 2:**
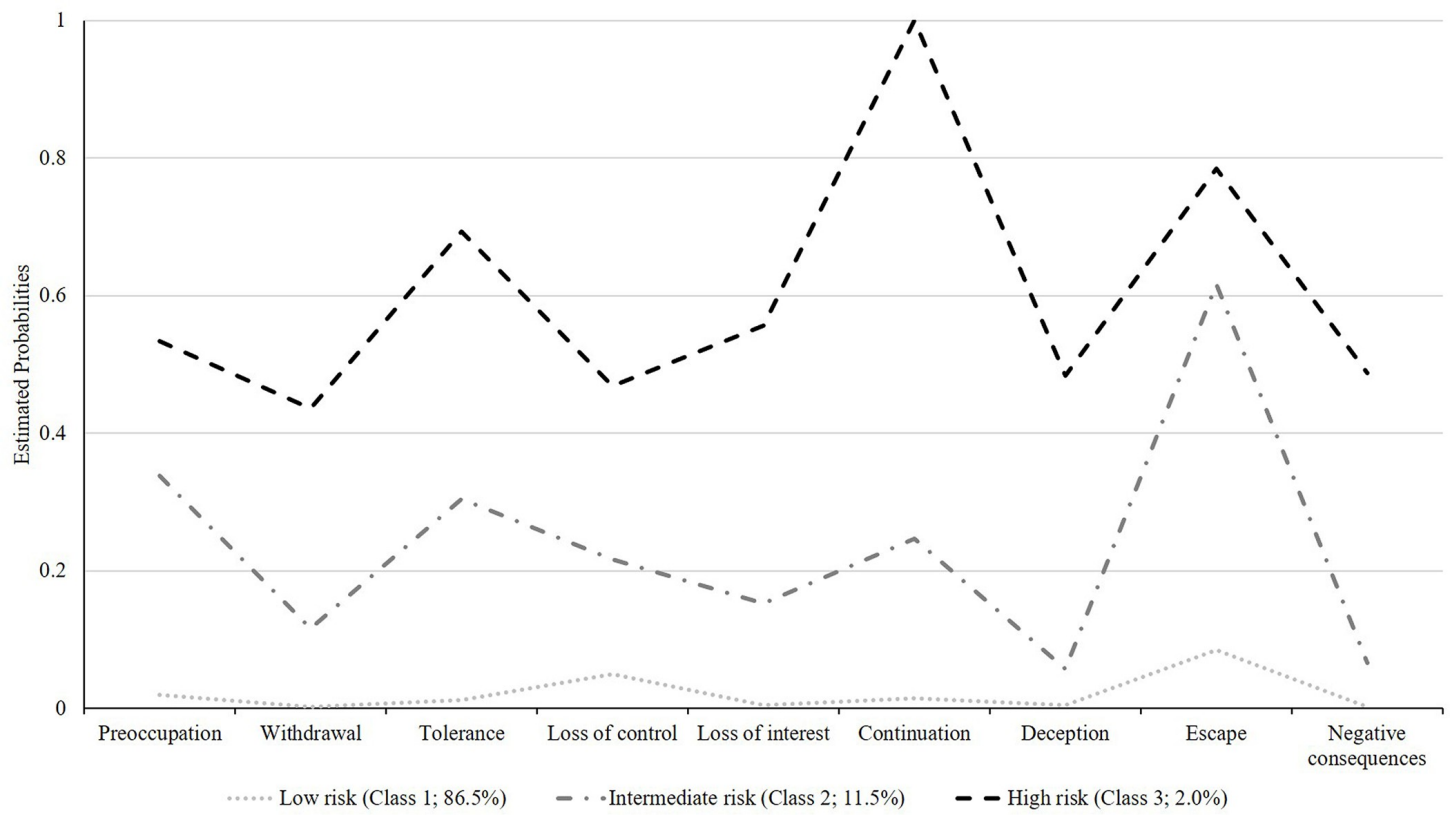
The 3-class solution of latent classes analysis of the nine IGD criteria from Indonesian IGDT-10.

Based on the LCA results, the 4-class model did not yield a significant LMR or bootstrap LRT. The 3-class solution had lower ABIC and AIC than the 2-class model (see Table 3). As shown in Fig 2, the first class, low risk IGD gamers (86.5%), had the lowest probability of endorsement in any of the criteria. The second class, the group with intermediate risk of IGD (11.5%), had moderate probabilities across domains and the third class, high-risk IGD gamers (2.0%), had the highest probabilities on all criteria. The escape domain emerged to significantly differentiate between classes 1 and 2. Classes 2 and 3 were predominantly discriminated by the continuation and negative consequence domains. Using the 5-point cutoff suggested by the original scale, this study detected a point-prevalence estimate of 1.90% (N = 24) among the 1265 respondents (inclusion of 32 respondents who did not play video games).

**Table 3:** Fit indices of the latent class analysis of the Indonesian IGDT-10.

Number of Latent Classes	BIC	ABIC	AIC	LMR	*P*	Bootstrap LRT *P*-value
**2-classes**	4458.93	4398.58	4361.71	655.33	< 0.001	< 0.001
**3-classes**	4486.94	4394.82	4338.54	43.17	0.034	< 0.001
**4-classes**	4537.58	4413.70	4338.01	20.53	0.22	0.43

**Fig 3:**
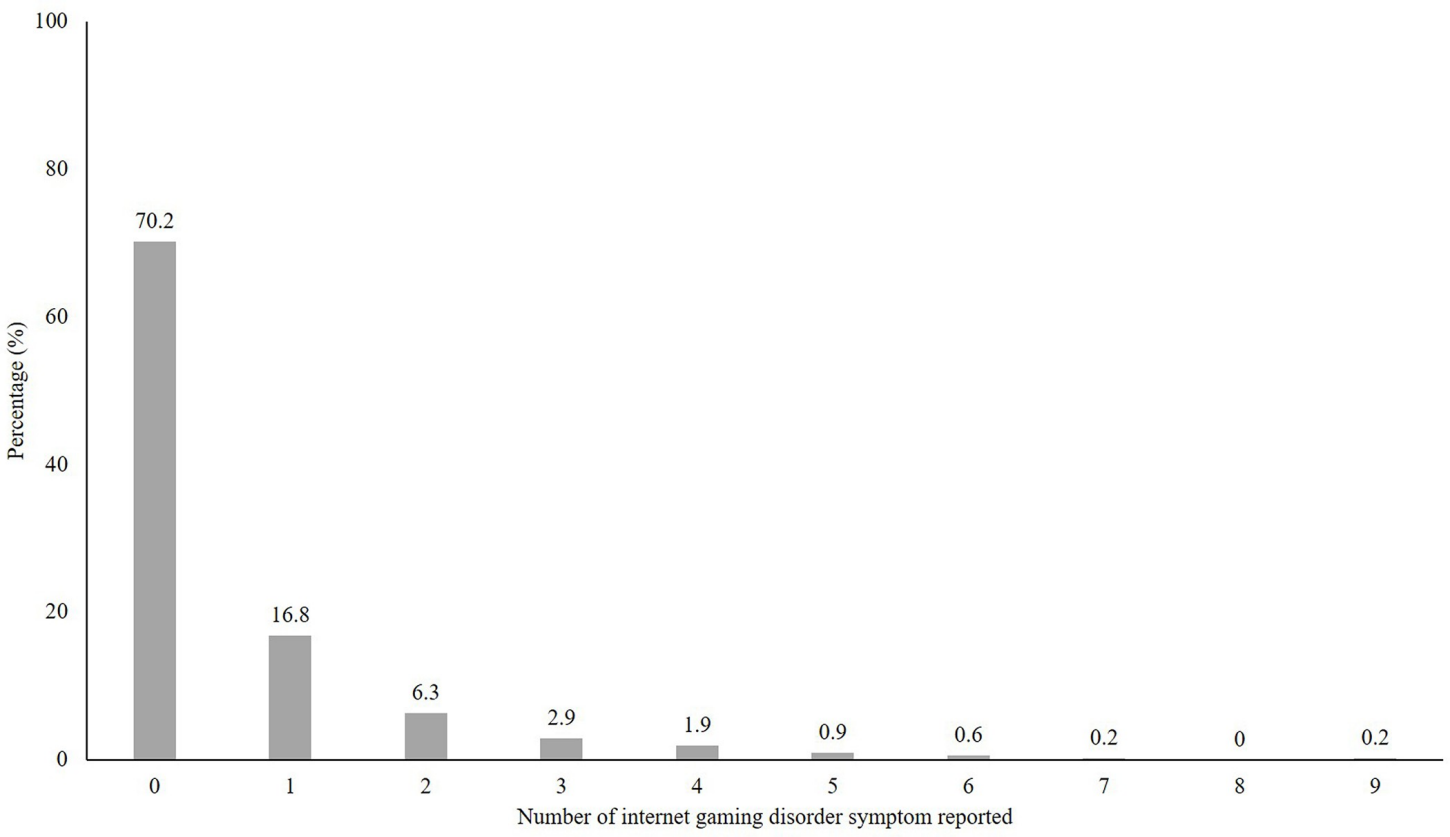
Distribution of reported frequencies of internet gaming disorder symptoms.

Fig 3 depicts the spread of the reported number of symptoms of IGD among the respondents: approximately 70.2% exhibited no symptoms, 16.8% exhibited one symptom, 6.3% exhibited two symptoms, 2.9% exhibited three symptoms, and 1.9% exhibited four symptoms. In Table 4, the rate across the nine constructs ranged from 41.7% (deception) to 91.2% (continuation) for IGD-risk group. The highest endorsement rate in the total sample was the escape domain, 17.8%.

**Table 4:** Endorsement of nine IGD criteria in the IGD-risk group and the total sample.

Criteria	Endorsement among the IGD^a^-risk respondents (N = 24)	Endorsement in the total sample (N = 1233)
	N (%)	N (%)
**1. Preoccupation**	16 (66.7)	94 (7.6)
**2. Withdrawal**	13 (54.1)	34 (2.8)
**3. Tolerance**	19 (79.1)	85 (6.9)
**4. Loss of control**	15 (62.5)	102 (8.3)
**5. Loss of interest**	13 (54.2)	47 (3.8)
**6. Continuation**	22 (91.2)	84 (6.8)
**7. Deception**	10 (41.7)	28 (2.3)
**8. Escape**	21 (87.5)	219 (17.8)
**9. Negative consequences**	12 (50.0)	26 (2.1)

^a^IGD, Internet gaming disorder

### Sociodemographic and gaming-related variables differences and correlation to IGDT-10

There were statistically significant differences, as shown in Table 5, in the duration of weekday (Cohen’s d = 2.05) and weekend gaming (Cohen’s d = 3.46) between IGD-risk and healthy respondents, with durations being nearly twice as long in the group with high risk of IGD. The correlation analysis demonstrated significant small to moderate links between IGDT-10 raw score with age (r = -0.067, *P* = 0.02), age of first gaming (r = -0.075, *P* = 0.009), weekday gaming duration (r = 0.34, *P*≤ 0.001), and weekend gaming duration (r = 0.35, *P≤* 0.001). The male sex had higher odds of IGD (OR = 3.33, 95% CI 1.02–10.90, *P* = 0.04, Cramer’s V = 0.29). Each category on the purpose of playing games, game genres, and negative consequences was compared to respondents who answered no. The results showed that respondents with achievement goals were at six times greater risk of IGD (OR = 6.60, 95% CI 1.25–34.95, *P* = 0.02, Cramer’s V = 0.35). Respondents who played Battle Royale had at least eleven times greater risk of experiencing IGD (OR = 11.50, 95% CI 1.31–101.18, *P* = 0.01, Cramer’s V = 0.37). There were significant differences between the IGD-risk and healthy respondents for poorer sleeping schedule (OR = 3.55, 95% CI 1.04–12.06, *P* = 0.04, Cramer’s V = 0.30) and work/academic related issues (OR = 9.31, 95% CI 1.78–48.72, *P =* 0.003, Cramer’s V = 0.42). Only 20.8% (N = 5) of the IGD-risk respondents indicated that they played offline games more often than they played online games. More than half (70.4%) accessed games through their mobile phones. The majority of the group at high risk of IGD relayed that their purpose of gaming was entertainment (66.7%). The most common game genre among the IGD-risk respondents was first-person shooting (33.3%) and battle royale (33.3%). Approximately 54.2% of the at-risk gamers claimed to suffer poor sleeping patterns and 45.8% further suffered from work or academic related issues.

**Table 5:** Sociodemographic profiles between IGD-risk and healthy respondents.

Variable		Healthy Respondents (N = 1209)	IGD^a^-risk Respondents (N = 24)	T/χ2^#^	Cohen’s d / Cramer’s V	Total (N = 1233)
		M±SD/N(%)	M±SD/N(%)			M±SD/N(%)
**Age**		20.3±1.9	20.7±1.6	0.67	1.74	20.3±1.9
**Age of first gaming**		10.9±3.6	10.5±4.4	-0.32	4.08	10.9±3.6
**Weekday gaming duration**		1.7±2.2	3.0±1.8	2.08*	2.05	1.7±2.2
**Weekend day gaming duration**		3.1±2.6	7.2±4.1	4.11***	3.46	3.1±2.7
**Sex**						
	**Male**	454 (37.6)	16 (66.7)	4.09*	0.29	470 (37.4)
	**Female**	755 (62.4)	8 (33.3)			763 (62.6)
**Residence**						
	**Without family**	546 (44.7)	9 (37.5)	0.34	0.09	555 (45.0)
	**With family**	663 (54.8)	15 (62.5)			678 (55.0)
**Gaming community**						
	**Yes**	172 (14.2)	8 (33.3)	2.95	0.25	180 (14.6)
	**No**	1037 (85.8)	16 (66.7)			1053 (85.4)
**Main Gaming platform**						
	**Mobile phones**	855 (70.7)	13 (54.2)	3.00	0.25	868 (70.4)
	**PC** ^**b**^ **/desktop**	118 (9.8)	6 (25.0)			124 (10.1)
	**Laptop**	178 (14.7)	4 (16.7)			182 (14.8)
	**Tablet**	58 (4.8)	1 (4.2)			59 (4.8)
**Purpose of playing games**						
	**Entertainment**	683 (56.4)	16 (66.7)	0.36	0.09	699 (56.7)
	**Pastime**	499 (41.2)	15 (62.5)	3.00	0.25	514 (41.7)
	**Relieve stress**	398 (32.9)	14 (58.3)	3.02	0.25	412 (33.4)
	**Gain achievement**	113 (9.3)	9 (37.5)	5.78*	0.35	122 (9.9)
	**Socialize**	191 (15.8)	8 (33.3)	2.95	0.25	199 (16.1)
**Game genres**						
	**First person shooting**	149 (12.3)	8 (33.3)	2.95	0.25	157 (12.7)
	**Battle Royale**	68 (5.6)	8 (33.3)	6.70**	0.37	76 (6.2)
	**Simulation games**	282 (23.3)	7 (29.2)	0.44	0.10	289 (23.4)
	**Sports**	150 (12.4)	7 (29.2)	2.02	0.21	157 (12.7)
	**Multiplayer online battle arena**	155 (12.8)	6 (25.1)	1.23	0.16	161 (13.1)
	**Real time strategies**	118 (9.7)	6 (25.0)	2.40	0.22	124 (10.1)
	**Massively multiplayer online role-playing games**	97 (8.0)	4 (16.7)	0.76	0.21	101 (8.2)
	**Puzzle**	359 (29.7)	4 (16.7)	1.06	0.15	363 (29.4)
	**Fighting**	94 (7.8)	4 (16.7)	0.76	0.13	98 (7.9)
**Negative consequences**						
	**Poor sleep patterns**	323 (26.7)	13 (54.2)	4.27*	0.30	336 (27.3)
	**Work/ academic related issues**	94 (7.8)	11 (45.8)	8.55**	0.42	105 (8.6)
	**Physical symptoms**	162 (13.2)	9 (37.5)	4.00	0.29	171 (13.9)
	**Poor eating patterns**	78 (6.5)	7 (29.2)	3.42	0.27	85 (6.9)
	**Mood related problems**	69 (5.7)	6 (25.0)	4.18	0.30	75 (6.1)
	**Relationship related problems**	37 (3.1)	6 (25.0)	4.18	0.30	43 (3.5)
	**Weight gain**	46 (3.8)	3 (12.5)	1.09	0.15	49 (4.0)
** **	**Hostility**	49 (4.1)	1 (4.2)	0	0	50 (4.1)

**p ≤ 0.01

***p ≤0.001

^a^IGD, Internet Gaming Disorder

^b^PC, Personal computer

^#^The results were the comparisons between the average of a hundred repeated randomizations of samples comprising 24 healthy respondents each and the IGD-risk group of a similar size (N = 24)

## Discussion

This study asserts that the Indonesian IGDT-10 was valid and reliable among Indonesian youths. The CFA yielded a satisfactory unifactorial structure of IGDT-10, consistent with the original study [10] and several other translated versions [12–14]. The unidimensional solution yielded sufficient factor loadings across items and acceptable goodness-of-fit indices. Additionally, the overall translated scale exhibited good internal reliability, compared to the original study [10]. These data suggest that the Indonesian IGDT-10 has a robust factor model.

Among the nine IGDT-10 criteria within IGD-risk group, the deception domain had the lowest endorsement; in contrast, continuation (despite harmful consequences) was the highest. The continuation and negative consequences domains also appeared to be distinctively endorsed by the high risk of IGD group (class 3), displaying the highest difference in LCA estimated probabilities compared to other IGD-risk groups’ values. Individuals with IGD have been noted to suffer from impaired cognitive control and maladaptive urge stimulation, particularly among youths [19], which would drive continued gaming despite negative consequences. Moreover, negative outcomes have been touted as a main domain that differentiate between healthy online behaviors (e.g., gaming or other internet activities) and pathological uses [20, 21]. Impairments and harms are also the hallmark of any mental disorder and prerequisite for diagnoses to be made [22, 23]. Interestingly, the extremely high endorsement rate of continuation and negative consequences among IGD-risk gamers have been reported by other studies on IGDT-10 as well [10, 13, 14], indicating shared commonalities across distinct cultures and regions.

The low endorsement of deception criteria is in line with previous studies [24, 25], indicating that deception, borrowed from problematic gambling [26], is not critically central to IGD. Notably, more than half of the respondents in this study reported living with their families and mainly played from their homes. There was evidence for the construct of deception, particularly for problematic gamers residing with family members. However, this is significantly reliant on the polarity of view of the parents or guardians, and how accommodating they are with games as a pastime [27]. Furthermore, some experts suggested that this construct could be more specifically reflective of problematic gamers suffering from conduct disorders [7].

Some related studies have noted a correlation between escapism and gaming. The former influenced many game-associated issues and mediated psychopathologies to gaming disorders [28]. The escape domain would be apparent in a population with concurrent high risk of IGD and high psychiatric and social distress [29]. Alternatively, the present study and several others [24, 30] indicated that gaming as a form of escape had lower probability than other domains, and that escaping dysphoric mood was also common among highly immersed gamers but with a low risk of IGD [30]. Analyzing the 3-class model from LCA in this study, the escape domain significantly separated between the low-risk group (class 1) and the group at moderate risk of IGD (class 2), but did not substantially distinguish between the group with highest risk of IGD (class 3) and the group with intermediate risk of IGD (class 2). Altogether, this suggests the importance of the item’s wording and is dependent on the respondents’ insight [7]. Additionally, this domain would prove helpful in differentiating healthy gamers from those at heightened risks and identify the subgroup at high risk of IGD with co-occurring psychiatric disorders.

This study screened 1.90% of the respondents with risk of IGD using the original 5-point cutoff. The LCA yielded a 3-class solution that encompassed a subpopulation with high risk of IGD (class 3) at a rate of 2.0%. Together, this indicates that the cutoff value of 5 is valid for use in Indonesian IGDT-10. The cutoff was consistent with the DSM-5 consensus and results of a clinical structured interview [12]. Additionally, this prevalence estimate was concordant with the global range of IGD among youths, such as Taiwanese at 3.1% [12], Japanese at 1.80% [31], Hungarians at 2.9% [10], and Finnish at 1.3% [13]. However, other studies in the Asian region have reported higher estimates ranging from 9.9–15.1% [4, 32, 33]. This could be attributed to sampling (non-random) bias and the use of tools with loose criteria that did not correspond to the requirements set out by either DSM-5 or ICD-11. However, with only over a tenth being detected to suffer a certain degree of problematic gaming and a smaller percentage met the requirements of IGD, this subpopulation of increased risk attested to the nature of the disorder as a spectrum and necessitated a multipronged approach toward IGD [34]. Moreover, this subpopulation would benefit from selective and indicated prevention, in contrast to a universal approach for the broader population of healthy gamers [35]. Consequently, the translated IGDT-10 would prove beneficial for screening in the general population and primary healthcare setting because it satisfies all criteria deemed significant by DSM-5 and ICD-11 [9].

Despite comprising mostly female respondents, this study demonstrated higher odds of IGD in male respondents, which was consistent with previous studies [6, 7]. The risk could be socially and biologically driven, especially because the larger gaming community is still predominantly male [36]. This study also indicates that the group at high risk of IGD played for significantly longer periods during weekdays and weekends. The IGDT-10 score in this study was found to have a positive correlation with both hours spent gaming per day during weekdays and weekends. This was consistent with the predominant continuation domain among the respondents at high risk of IGD. Interestingly, a fifth of the IGD-risk respondents had mainly played offline games, which supported the inclusion of both online and offline games. However, the online platform seemed more predominant, which corresponds with previous findings [29, 37].

Overall, to the best of the authors’ knowledge, this study is the first to validate an IGD instrument in the Indonesian language and employs the largest number of respondents. This study indicates that the Indonesian IGDT-10 is psychometrically sound and has a prevalence estimate of 1.90%, concordant with the global range of IGD. However, this study has some limitations. First, this study employs a convenience sample by recruiting only youths from two cities and universities in Indonesia, which might hamper its generalizability. Second, the instrument was self-reported and might have suffered from social desirability bias. Third, there is a lack of sociodemographic profiles (e.g., female was more represented and links to faculties’ differences were not explored) and the gaming console data (e.g., PlayStation, Xbox) were not provided as response options. Further, the possible data hierarchies and relationships between nested levels were not accounted for during sampling, which could introduce biased estimates. Fourth, psychological risk factors were not assessed while testing the construct validity of the instrument. Lastly, this study neither include test-retest reliability nor compare the IGDT-10 instrument with the gold standard (i.e., psychiatric interview); therefore, the values of accuracy, specificity, and sensitivity could not be determined. Further studies should be conducted to facilitate the comparison.

## Conclusion

The Indonesian version of the IGDT-10 was validated as a one-factor structure model among a sample of youths, which was similar to the original study and other translated versions. The translated items demonstrated satisfactory internal reliability. The LCA yielded three distinct groups of gamers, with the high risk of IGD (class 3) subpopulation’s rate conforming to the detected IGD prevalence estimate of 1.90% among Indonesian youths. Continuation and negative consequences criteria displayed the highest information to determine the group at high risk of IGD. Similar to previous studies, the deception and escape domains had the lowest probability. There was also evidence of association between IGD and male sex and gaming duration during weekdays and weekends.
